# Chemometric and Transcriptomic Profiling, Microtubule Disruption and Cell Death Induction by Secalonic Acid in Tumor Cells

**DOI:** 10.3390/molecules25143224

**Published:** 2020-07-15

**Authors:** Nadire Özenver, Mona Dawood, Edmond Fleischer, Anette Klinger, Thomas Efferth

**Affiliations:** 1 Department of Pharmacognosy, Faculty of Pharmacy, Hacettepe University, 06100 Ankara, Turkey; nadire@hacettepe.edu.tr; 2 Department of Pharmaceutical Biology, Institute of Pharmacy and Biomedical Sciences, Johannes Gutenberg University, Staudinger Weg 5, 55128 Mainz, Germany; modawood@uni-mainz.de; 3 Department of Molecular Biology, Faculty of Medical Laboratory Sciences, Al-Neelain University, Khartoum 11121, Sudan; 4 MicroCombiChem GmbH, 65203 Wiesbaden, Germany; edmond.fleischer@gmx.de (E.F.); anette.klinger@microcombichem.de (A.K.)

**Keywords:** cancer, drug resistance, microarray analysis, multiple myeloma, mycotoxins

## Abstract

Nature is an indispensable source of new drugs, providing unique bioactive lead structures for drug discovery. In the present study, secalonic acid F (SAF), a naturally occurring ergochrome pigment, was studied for its cytotoxicity against various leukemia and multiple myeloma cells by the resazurin assay. SAF exhibited cytotoxic activity on both leukemia and multiple myeloma cells. Generally, multiple myeloma cells were more sensitive to SAF than leukemia cells. NCI-H929 cells were the most affected cells among the tested panel of multiple myeloma cell lines and were taken for further studies to assess the mode of action of SAF on those cells. Cell cycle analysis revealed that SAF induced S and G2/M arrest in NCI-H929 cells. SAF-associated apoptosis and necrosis resulted in cytotoxicity. SAF further inclined the disassembly of the tubulin network, which may also account for its cytotoxicity. COMPARE and hierarchical cluster analyses of transcriptome-wide expression profiles of the NCI tumor cell line panel identified genes involved in numerous cellular processes (e.g., cell differentiation, cell migration, and other numerous signaling pathways) notably correlated with log_10_IC_50_ values for secalonic acid. In conclusion, the present study supports the therapeutic potential of SAF to treat multiple myeloma.

## 1. Introduction

Cancer, the second leading cause of death globally, caused 9.6 million deaths in 2018 worldwide [1]. Currently, the 5-year survival rate of patients receiving cancer therapy is 67% [2,3]. In 2014, 14.5 million cancer patients survived their disease in the United States and the number of cancer survivors is considered to reach 19 million by 2024 [4]. The development of effective and personalized drugs with attenuated side effects holds significant prominence for drug discovery against cancer.

Multiple myeloma (MM) is an incurable disease leading 1% of all cancers and 10% of hematological malignancies with low survival rates worldwide [5,6]. The mean survival of patients with MM is nearly 2.5 to 3 years, and the 10-year survival rate is around 30% under 60 years of age [7,8,9,10]. Recently, newly developed drugs such as proteasome inhibitors, immunomodulatory drugs, corticosteroids, and alkylating agents have extended patient survival up to 5–6 years following diagnosis [9,11]. Novel agents targeting various proteins and signal transduction pathways are under investigation to overcome the low survival rates of MM patients.

Leukemia, characterized by an increased number of leucocytes in the blood and/or the bone marrow, is another entity of life-threatening hematological malignant disorders [12]. Leukemia is the most prevalent cancer in children younger than 15 years of age and also occurs in adults older than 55 years [13]. Acute lymphoblastic leukemia (ALL) and acute myeloid leukemia (AML) are among the most common types of leukemia. About 80% of ALL develop in children [14]. Despite a high grade of chemotherapy response, only 30–40% of adult patients with ALL will accomplish long-term remission [15]. Likewise, AML is cured in 35 to 40% of adult patients, who are 60 years of age or younger, and in 5–15% of patients, who are older than 60 years of age [16]. Therefore, new therapeutic agents with minimal toxicities are required to prolong the survival of patients with MM or leukemia.

Nature represents an indispensable source providing bioactive natural products with potential for cancer therapy. To exemplify, only 13% of the approved anticancer drugs from 1981 to 2014 were totally synthetic drugs. The others include biological macromolecules (19%), unaltered natural products (10%), botanical drugs (1%), natural product derivatives (22%), synthetic drugs (mimics of natural products, 11%), synthetic drugs (the pharmacophore was from natural product, 7%), synthetic drugs (the pharmacophore was from natural product mimic, 14%) and vaccines (3%). Besides, apart from non-biologicals/vaccines, there were 136 new chemical entities in the time frame covered (January 1981–December 2014) in the field of anticancer drugs, 113 (83%) of which were either natural products, natural product derivatives or natural product mimics [17]. Natural products are either used by local people in traditional medicine or provide attractive opportunities for drug discovery as lead compounds, in order to find (semi) synthetic derivatives with improved pharmacological features due to a wide scale of the index of highly selective and effective compounds.

Secalonic acids, a series of ergochrome pigments, are secondary metabolites isolated from fungi such as *Claviceps purpurea*, *Aspergillus ochraceus*, *Aspergillus aculeatus*, *Pyrenochaeta terrestris*, and *Penicillium oxalicum* [18,19,20,21,22,23]. Structurally, they mainly comprise the dimers of six different monoxanthones, which are linked at the 2,2′ positions with equal molecular weights (638.1635) and have the molecular formulae (C_32_H_30_O_14_), despite their different stereochemistry at positions 5, 6, 10a, 5′, 6′, and 10a’(secalonic acids A–F) [21,22,23,24].

**Figure 1:**
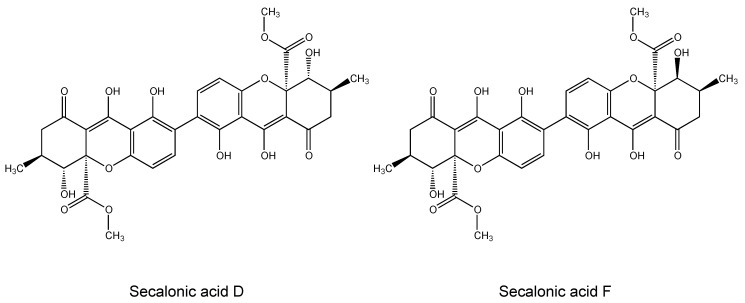
The chemical structures of secalonic acid D and secalonic acid F.

Secalonic acids have been reported to exert various biological activities including antibacterial, antiphlogistic, and antitumor features [19,24,25,26,27]. In particular, a number of studies have unraveled the cytotoxic properties of secalonic acid derivatives on numerous cancer cell lines [28,29,30,31]. Secalonic acid D (SAD) (Figure 1), a widely investigated member of the group, is well known for its anticancer properties by acting as DNA topoisomerase 1 inhibitor [32]. SAD also demonstrated remarkable cytotoxic activity on murine leukemia P388 cells, human chronic myeloid leukemia K562 cells, human lung carcinoma A549 cells under the general culture conditions as well as on human pancreatic carcinoma PANC-1 cells under glucose-starved conditions [33,34]. In addition to its effect on chemotherapy-sensitive cells, SAD caused potent cytotoxicity toward ABCB1-, ABCC1-, or ABCG2- overexpressing multidrug-resistant cancer cells [35]. Furthermore, SAD induced apoptosis and activated cell death signaling cascades in assorted cell lines [36,37,38]. Unfortunately, despite having anticancer properties, SAD reveals considerable toxicity, which makes its clinical use difficult [39].

Secalonic acid F (SAF) (Figure 1), another member of the secalonic acid family, is originally obtained from *Claviceps purpurea* [40]. SAF acts as an allelochemical [41,42] and anti-tumor agent suppressing the growth and progression of hepatocellular carcinoma cells both in vitro and in vivo by affecting several pathways such as the activation of an intrinsic mitochondria-mediated apoptotic pathway [43] or inactivation of PI3K/AKT/ β-catenin signaling [44]. In another study, SAF inhibited HL-60 cell proliferation in a dose- and time-dependent manner and induced apoptosis [45].

Despite the presence of many studies emphasizing the antitumoral properties of the secalonic acid family, studies on SAF have been quite limited thus far. In the present study, we investigated the function of SAF in various cancer cells from different origins and focused on the understanding of molecular mechanisms underlying its cytotoxicity. Second, we addressed the question of whether the cytotoxicity of SAF was associated with other molecular determinants in the cell line panel of the National Cancer Institute (NCI, Bethesda, MD, USA). Moreover, we performed bioinformatic COMPARE and hierarchical cluster analyses of microarray-based transcriptomic mRNA expression data of the NCI cell lines that were correlated to SAF’s response.

SAF does not only hold promise as a naturally occurring effective and selective agent, but also serves as model for the development of (semi) synthetic drug leads in cancer therapy. Therefore, it requires further investigations in the field of cancer.

## 2. Results

### 2.1. Cytotoxicity of Secalonic Acid F toward Leukemia and Multiple Myeloma Cells

**Figure 2:**
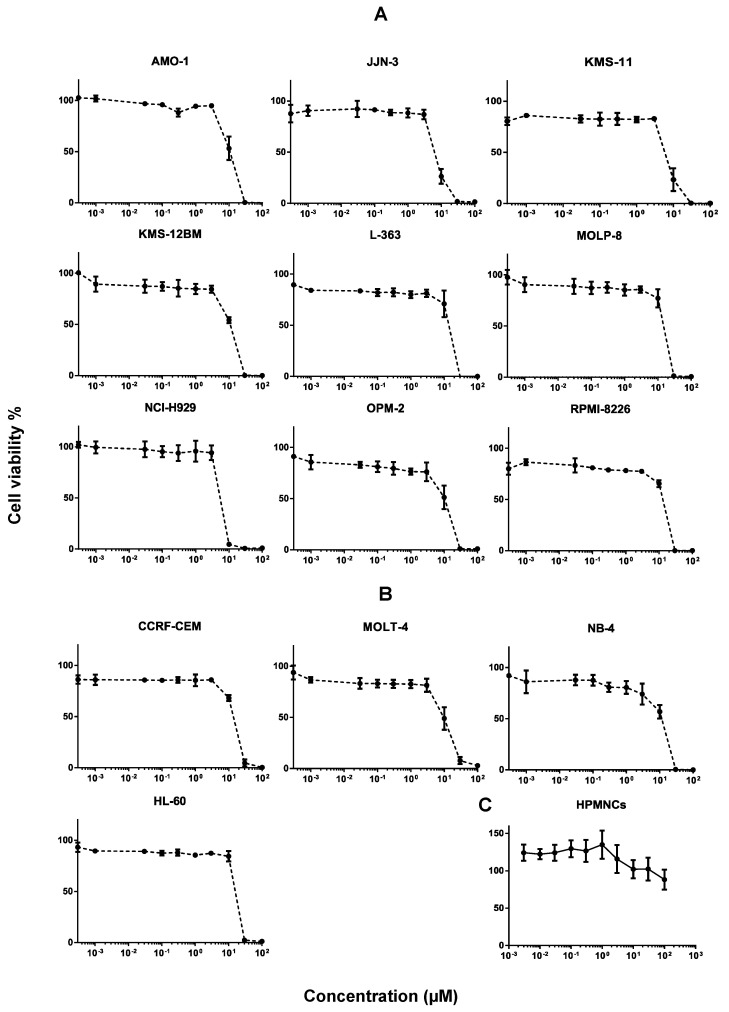
Dose-response curves of secalonic acid F (SAF). (**A**) Cytotoxicity of SAF toward various multiple myeloma cells as determined by the resazurin assay. (**B**) Cytotoxicity of SAF toward various leukemia cells as determined by the resazurin assay. (**C**) Toxicity of SAF toward human peripheral mononuclear cells (HPMNCs) as determined by the resazurin assay.

The cytotoxicity of SAF was tested in different cancer cells and dose-response curves were created (Figure 2A,B). SAF exhibited 50% cell viability inhibition in leukemia cells of CCRF-CEM, HL-60, MOLT-4, and NB-4 at the concentrations of 15.89 ± 0.73, 18.35 ± 0.62, 10.75 ± 3.02, and 12.29 ± 2.09, respectively. Meanwhile, SAF exerted 50% inhibition of cell viability in AMO-1, JJN-3, KMS-11, KMS-12BM, L-363, MOLP-8, NCI-H929, OPM-2, and RPMI-8226 in MM cells at concentrations of 11.51 ± 3.10, 7.28 ± 0.71, 6.99 ± 0.77, 11.51 ± 1.04, 15.66 ± 2.66, 17.06 ± 1.69, 6.44 ± 0.31, 10.68 ± 3.29, and 14.72 ± 0.78, respectively. Generally, these results indicate that MM cells were more sensitive to SAF than leukemia cells. Particularly, SAF displayed the strongest cytotoxicity on NCI-H929 among other MM cells.

### 2.2. Toxicity of Secalonic Acid F in Healthy Cells

SAF did not exhibit any toxicity on the human peripheral mononuclear cells (HPMNCs) at the IC_50_ concentrations of the tested MM and leukemia cell lines. It was not toxic enough to determine its IC_50_ on HPMNCs (IC_50_ > 100 µM), and even then the cell viability did not drop below 50% at the highest concentration we studied (100 µM). Furthermore, nearly 100% cell viability was observed at the IC_50_ of NCI-H929 (Figure 2C).

### 2.3. Cross-Resistance of Secalonic Acid to Established Anticancer Drugs

**Figure 3:**
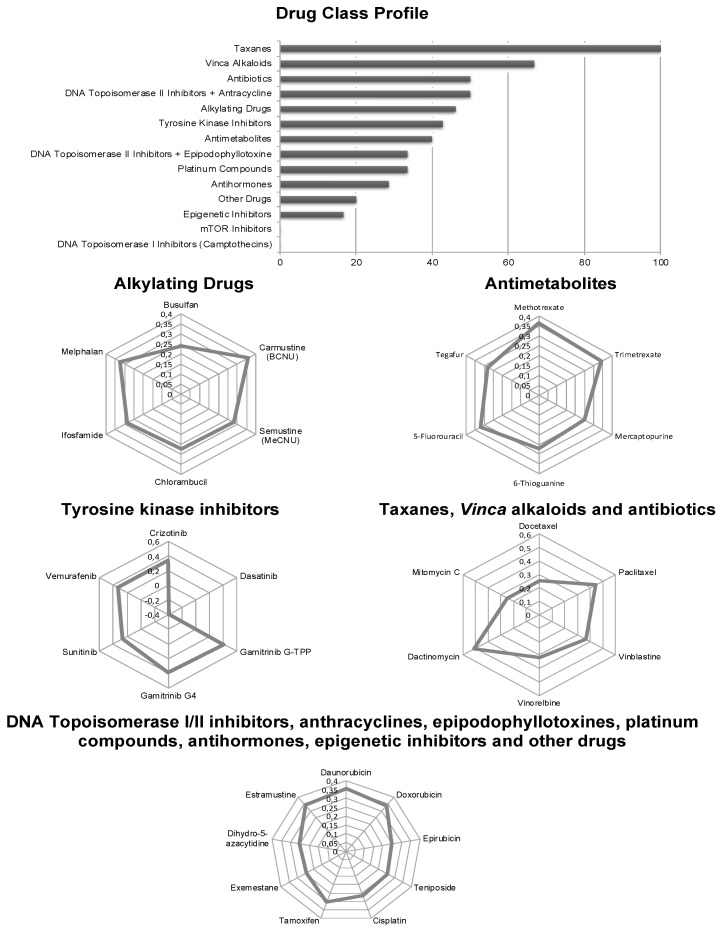
Oncobiograms of the cross-resistance profiles of the National Cancer Institute (NCI) cell line panel between secalonic acid and different classes of established anticancer drugs.

We correlated the log_10_IC_50_ values of the NCI cell lines to secalonic acid with those of 90 standard drugs to obtain an idea of the probable modes of actions of secalonic acid. The cellular responses of taxanes significantly correlated with those of secalonic acid (100%). Two out of the three *Vinca* alkaloids (66.7%) significantly correlated with those of secalonic acid. Antibiotics (50%) as well as DNA topoisomerase II inhibitors and anthracyclines (50%) were further correlated with secalonic acid (*R* > 0.2 and *p* < 0.05). Alkylating drugs (6/13 = 46.2%), tyrosine kinase inhibitors (6/14 = 42.9%), and antimetabolites (6/15 = 40%) exerted comparable results. Reasonable outcomes were obtained for DNA topoisomerase II inhibitors and epidopodophyllotoxines (33.3%), platinum compounds (33.3%), antihormones (28.6%), other drugs (20%), and epigenetic inhibitors (16.7%). On the other hand, significant correlations were not found for mTOR inhibitors and DNA topoisomerase I inhibitors (Figure 3). These outcomes may imply that secalonic acid demonstrates multiple modes of action, a usual character of other phytochemicals.

### 2.4. Tumor-Type Dependent Response toward Secalonic Acid

**Figure 4:**
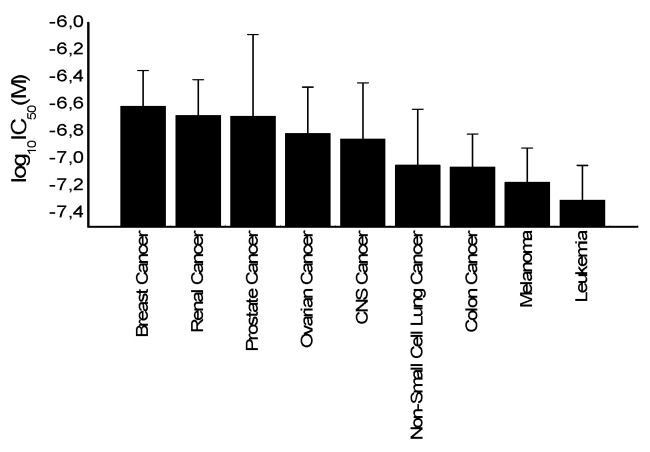
Mean values and standard deviations of log_10_IC_50_ values for secalonic acid of the NCI cell lines of different tumor types.

If the average of log_10_IC_50_ values of all cell lines was plotted based on their corresponding tumor type, leukemia cells appeared as the most sensitive tumor type toward secalonic acid, while breast cancer cells were the most resistant (Figure 4).

### 2.5. Microarray-Based Expression Profiling to Predict Sensitivity and Resistance to Secalonic Acid

We perused the transcriptome-wide mRNA expressions of the NCI cells and correlated them to the log_10_IC_50_ values for secalonic acid to find out the possible genes associated with sensitivity or resistance of cancer cells toward secalonic acid. We performed a transcriptome-wide COMPARE analysis to create a ranking list of genes, whose mRNA expression directly or inversely correlated with the log_10_IC_50_ values for secalonic acid. Only cut-off values of correlation coefficients of R > 0.4 (direct correlations) or R < 0.4 (inverse correlations) were considered. Forty genes were identified, among which half were directly and the other half were inversely correlated to the log_10_IC_50_ values for secalonic acid (Table 1). The proteins encoded by these genes exert numerous biological effects as indicated in Table 1.

**Figure 5:**
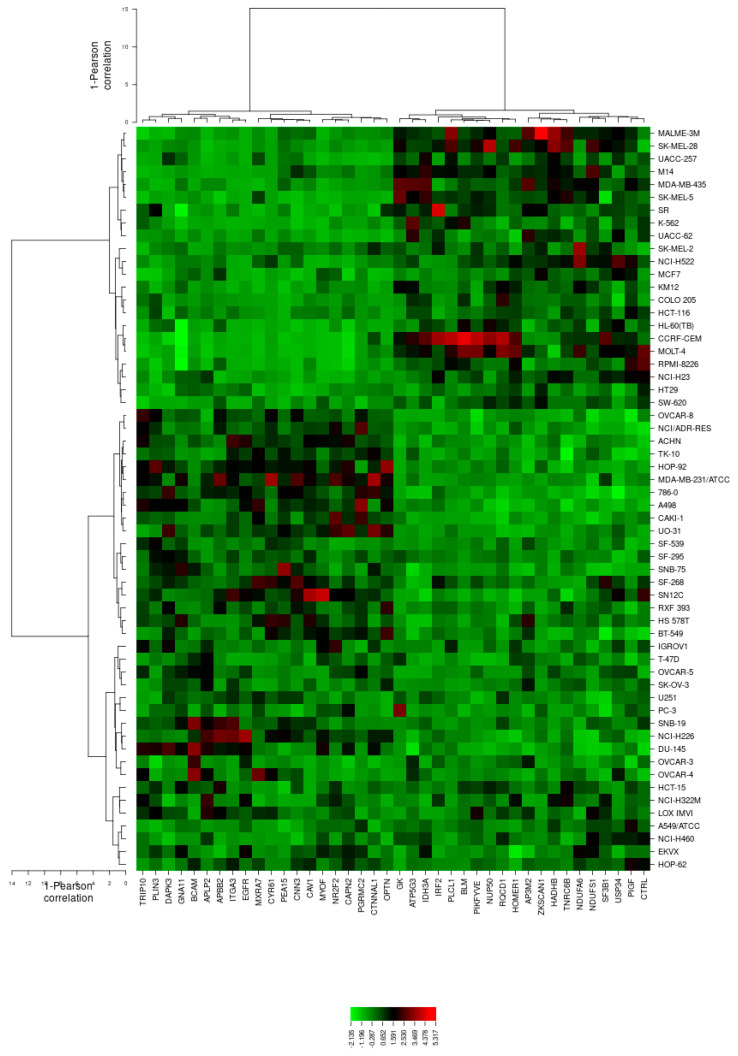
Dendrograms and heatmap of secalonic acid obtained by hierarchical cluster analyses of the NCI cell line panel and genes whose mRNA expression is directly or inversely correlated with the log_10_IC_50_ values for secalonic acid. The red frames on the dendrogram show the clustering of cell lines and the dendrogram on the top shows the clustering of genes.

Subsequently, the mRNA expression values of all NCI cell lines for the genes in Table 1 were submitted to hierarchical cluster analysis in order to ascertain whether clusters of cell lines could act in an identical manner prior to the secalonic acid treatment. The dendrogram of the cluster analysis pointed out four main branches in the cluster tree that were rendered in the heatmap (Figure 5). Subsequently, the log_10_IC_50_ values for secalonic acid, which were not included in the cluster analysis, were assigned to the corresponding position of the cell lines in the cluster tree. The distribution among the four clusters was significantly different from each other (*p* = 4.29 × 10^−4^). Clusters 3 and 4 were mostly comprised of cell lines resistant to secalonic acid, whereas clusters 1 and 2 generally included sensitive cell lines (Table 2). The median value of the log_10_IC_50_ values was used as a cut-off value to describe the cell lines as being sensitive or resistant to secalonic acid.

### 2.6. Cell Cycle Distribution and Apoptosis

**Figure 6:**
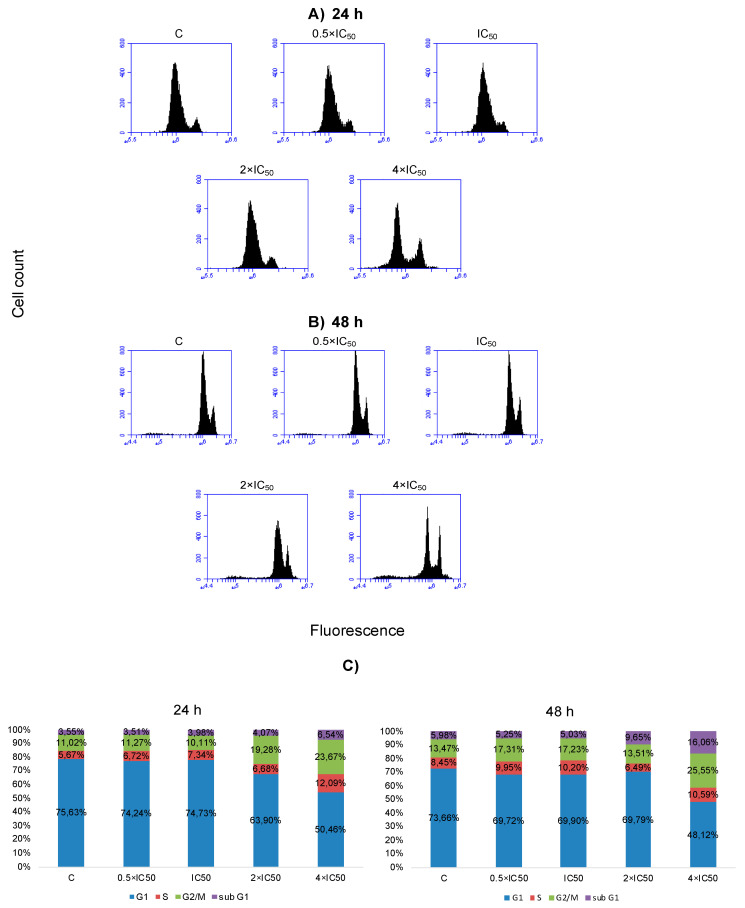
(**A**) DNA histograms of NCI-H929 cells treated with indicated concentrations of secalonic acid F (SAF) for 24 h, (**B**) DNA histograms of NCI-H929 cells treated with indicated concentrations of SAF for 48 h, and (**C**) Cell cycle distribution of NCI-H929 cells treated with indicated concentrations of SAF for 24 h and 48 h, respectively.

**Figure 7:**
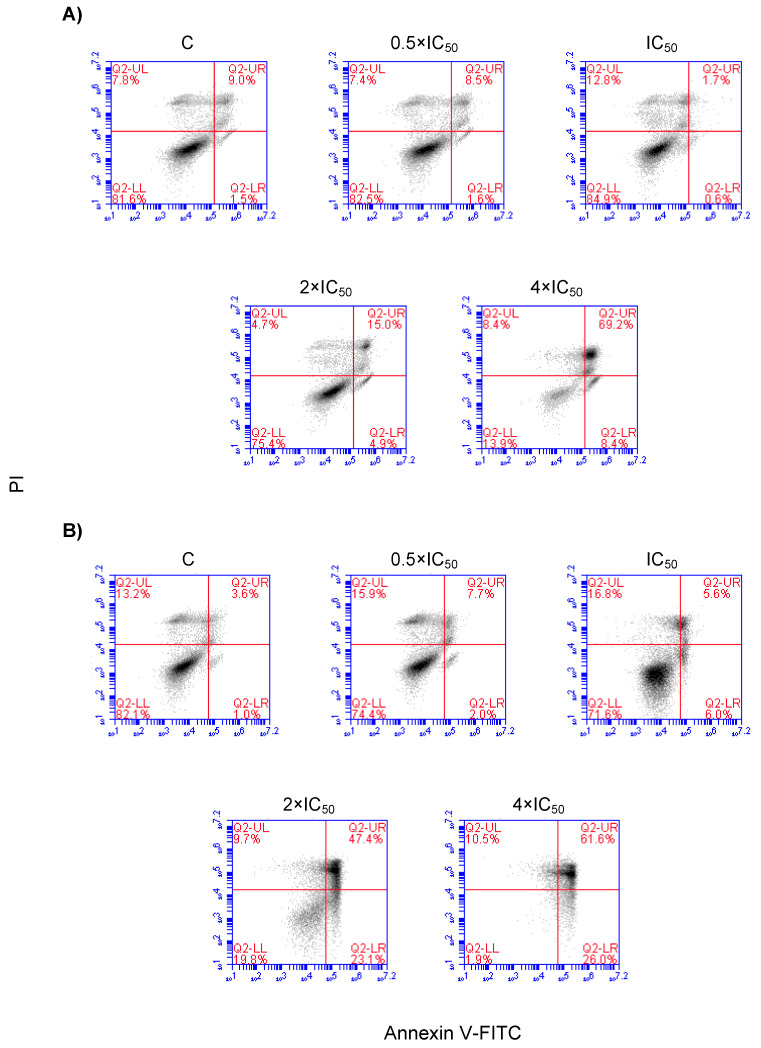
Apoptosis and necrosis effect in NCI-H929 cells treated with secalonic acid F (SAF) for 24 h (**A**) and 48 h (**B**).

SAF was used to treat NCI-H929 cells at various concentrations (0.5 × IC_50_, 1 × IC_50_, 2 × IC_50_, and 4 × IC_50_) followed by cell cycle analysis after 24 h and 48 h, respectively. The effect of SAF on the cell cycle distribution of NCI-H929 cells are depicted in Figure 6. SAF induced cell cycle arrest in the S and G2/M phases of this cell line upon treatment for 24 h, and 48 h at the concentrations of 2 × IC_50_ and 4 × IC_50_ (Figure 6). Induction of apoptosis/necrosis was further observed by annexin V/PI staining. The treated cells underwent both apoptosis and necrosis. Gated cells stated different cell populations equal to viable and non-apoptotic (annexin V−/PI−), early apoptotic (annexin V+/PI−), late apoptotic, and early necrotic (annexin V+/PI+) as well as late necrotic (annexin V−/PI+). SAF induced early and late apoptosis in addition to early and late necrosis at indicated concentrations (Figure 7).

### 2.7. Influence of Secalonic Acid F (SAF) on Microtubules

**Figure 8:**
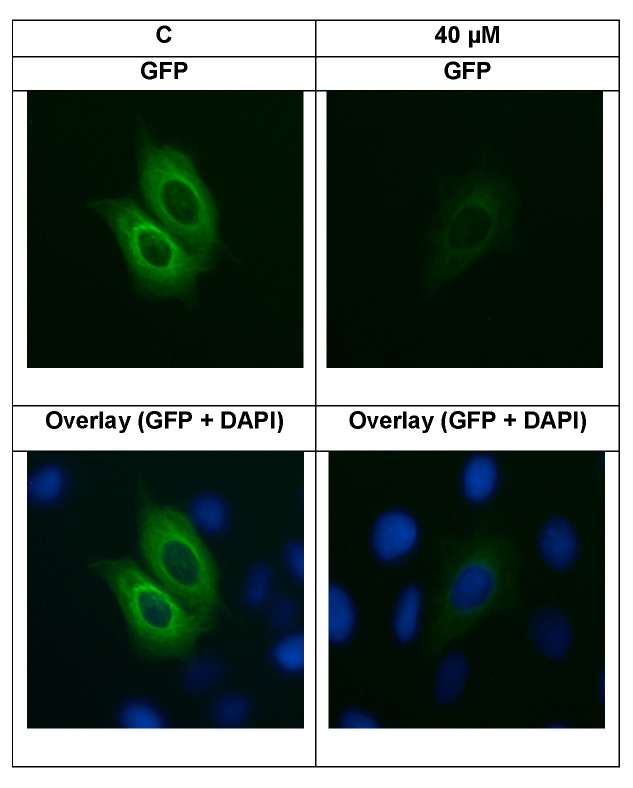
Secalonic acid F (SAF) altered the morphology of the microtubule network in U2OS cells. Panels show the micrographs of U2OS cells treated for 24 h at 40 µM of SAF.

The U2OS cells expressing α-tubulin-GFP were treated with SAF to assess its effect on the cellular microtubule network. In non-treated cells, microtubules continuously spread thorough the cytoplasm and generated an intracellular network. Treatment with SAF reduced the microtubule mass, presenting a decreased intensity at the cell periphery in comparison to the non-treated cells (Figure 8).

## 3. Discussion

### 3.1. Cytotoxicity of Secalonic Acid F toward Leukemia and Multiple Myeloma Cells

The outcomes of the resazurin assay showed that SAF was cytotoxic on both leukemia and MM cells, in particular, to the MM cells. NCI-H929 cells were the most sensitive cells among the other tested MM cells, and thus were taken for use in further studies to assess the mode of action of SAF on those cells. Despite several secalonic acid derivatives with different stereochemistry, SAF has rarely been studied in terms of cytotoxic and/or antitumor activities unlike some secalonic acid family members such as SAD. For instance, a study conducted by Gao et al. (2017) established that SAF exhibited higher cytotoxic activity than that of the antitumor drug fluorouracil on hepatocellular carcinoma cells in vitro and tumor growth in vivo. Cell cycle arrest and caspase-dependent apoptosis were demonstrated as underlying mechanisms regarding its cytotoxicity [43]. Li et al. (2012) studied the influence of SAF on HL-60 and indicated that SAF acted as a cytotoxic agent by inducing apoptosis and caspase 3-dependent RhoGDI 2 cleavage [45]. In a previous study, SAD induced apoptosis in HL-60 cells through cell cycle arrest of the G1 phase associated with the downregulation of c-Myc. HL-60 cells treated with SAD expressed RhoGDI 2 [37]. Li et al. (2012) observed the same altered profiles of RhoGDI 2 in HL-60 cells during SAF treatment, inferring that both SAF and SAD could lead to apoptosis through caspase 3 cleavage of RhoGDI 2 [37,45]. There has been a small number of studies about the cytotoxic activity of SAF and the comparison of SAF and SAD in terms of their cytotoxicities. To the best of our knowledge, this is the first comprehensive study on the cytotoxicity and mechanism of action of SAF in multiple myeloma cells. Based on the representative studies and their highly similar structures as isomer compounds, we believe that a mutual or comparative relationship between SAF and SAD may exist and that they can act in a similar manner to represent their cytotoxicity. Still, further studies are required to prove this statement.

### 3.2. Toxicity of Secalonic Acid F in Healthy Cells

The fact that SAF revealed virtually no toxicity on PMNCs despite its potent antitumor activity on leukemia and MM cells shows that it is a selective and potent antitumor agent. SAD had remarkable antitumor properties in numerous cancers [33,34,35,36,37,38]. However, it has been reported to have toxic effects on laboratory animals [46]. Therefore, SAF, with an almost identical structure to SAD, may represent an alternative and promising drug candidate molecule in cancer therapy.

### 3.3. COMPARE and Cluster Analyses of Microarray Data

COMPARE and cluster analyses of microarray data have pointed out that cell lines of different origin exhibited different degrees of sensitivity to secalonic acid (Figure 5). Among the various cancer cells from the different origins in the NCI database, leukemia cells were represented as the most sensitive (Figure 4). Likewise, our in vitro experimental outcomes confirmed the case exhibiting cytotoxicity on leukemia cells, but not on breast cancer cells. If SAF was tested toward a pair of wild-type and multidrug-resistant breast cancer cells (MDA-MB-231-pcDNA3 and MDA-MB-231-BCRP clone 23), it did not exhibit any cytotoxicity up to 100 µM (data not shown), confirming our assessments based on grouping the mean log_10_IC_50_ values for secalonic acid of the NCI cell lines according to the tumor type of the cell lines. From this point of view, we further studied SAF toward nine pairs of MM cells due to their similar hematopoietic origin to leukemia cells. Broadly, SAF was even more cytotoxic on the tested MM cells, among which NCI-H929 cells were the most sensitive. Therefore, we preferred to continue our investigations on MM cells to clarify the mode of action of SAF on those cells.

COMPARE analysis discovered the association between the gene expression patterns and the drug responsiveness measured by the NCI developmental therapeutic program. The mechanism of drug action, resistance, and sensitivity determinants may be predicted by the patterns of drug activity across the 58 NCI cell lines [47]. The procedure of specifying genes, whose mRNA expression correlated with secalonic acid’s activity, depends on the Pearson correlation coefficient. These correlation coefficients (Table 1) were calculated for each combination of a gene and a drug by taking the normalized level expression of the gene in each cell line, multiplying it by the corresponding (normalized) sensitivity of the cell line to the drug, summing the results overall of the cell lines and renormalizing [48]. Probable gene–drug interaction was examined through the combination of secalonic acid’s activity data with genome-wide expression profiling. Thus, this method enabled us to evaluate the data of a lot of genes and to make assumptions about the potential mode of action [49].

The main idea of cluster analysis was to group cell lines with respect to the levels of gene expressions correlated to the secalonic acid’s activity. The log_10_IC_50_ values were omitted from the calculation. Among the cell lines in the Developmental Therapeutics Program (DTP) of the NCI database, leukemia cells were clustered as the most sensitive ones, as shown in Figure 5. These prognostic findings were confirmed by our in vitro cytotoxicity outcomes, in which the smaller IC_50_ values were acquired on leukemia cells following secalonic acid treatment in comparison to the tested breast cancer cells.

### 3.4. Cross-Resistance Pattern of the National Cancer Institute (NCI) Cell Line Panel between Secalonic Acid and Standard Drugs

If we carried out a correlation analysis between the log_10_IC_50_ values of secalonic acid and those of the 90 standard anticancer drugs to describe the possible modes of action of secalonic acid, taxanes and *Vinca* alkaloids as microtubule -binding agents (MBAs) exhibited the highest correlation rates (100% and 66.7%, respectively). MBAs comprise either microtubule-stabilizing (e.g., taxanes) or microtubule-destabilizing (e.g., *Vinca* alkaloids) agents binding to novel sites on tubulin. Both classes of drugs potently inhibit microtubule dynamics and block mitosis [50]. MBAs can change the dynamic of mitotic spindles during mitosis, which may initiate the cell cycle checkpoint and thus arrest the cell cycle in the G2/M phase [51]. Taxanes are a class of anticancer agents, which bind to the *β*-tubulin subunit of microtubules causing the stabilization of microtubules and the disruption of microtubule function [50,52]. Therefore, we treated the U2OS cells expressing α-tubulin-GFP with SAF and analyzed the direct influence of SAF on the microtubule cytoskeleton by digital inverted microscopy to disclose the SAF influence on the microtubules. We observed reduced numbers of tubulin filaments as well as reduced intensity of tubulin staining (Figure 8) and concluded that the disassembly of the tubulin network may be one of the reasons accounting for secalonic acid-associated cytotoxicity. Furthermore, microtubules have an essential role in the directional migration of cells [53]. Since, our gene expression profiling demonstrated a number of deregulated genes (e.g., *PIKFYVE*, *ZKSCAN1*, *CYR61*, *ITGA3*) associated with the microtubule cytoskeleton, we assumed that secalonic acid’s effect on microtubules may be related to its ability to inhibit cancer cell migration.

### 3.5. Cell Cycle Distribution and Apoptosis/Necrosis

We performed cell cycle analysis to prove our hypothesis of whether the alterations in the dynamic of the mitotic spindle would induce G2/M arrest. Gene expression profiling also indicated that some deregulated genes (e.g., *IRF2*, *DAPK3*) were involved in cell cycle regulation. Our data revealed a direct relationship between cytotoxicity and cell cycle arrest in SAF-treated cells (Table 1, Figure 6). Cell cycle analysis of NCI-H929 cells unraveled both S and G2/M after SAF treatment at 24 h and 48 h time points, confirming our assumption that the inhibition of tubulin polymerization may result in cell cycle arrest.

If annexin V/PI staining was performed by flow cytometry, both apoptosis and necrosis were identified as mechanisms accounting for SAF’s cytotoxicity. Furthermore, deregulated genes associated with apoptosis (e.g., *PEA15* and *DAPK3*) validated the association of the cytotoxicity of SAF with apoptotic proteins and their affected pathways.

With regard to the COMPARE analysis, other genes from diverse functional groups appeared in our analysis such as cell differentiation (e.g., *RQCD1*) and other numerous signaling pathways (e.g., *PLCL1*, *USP34*, *TRIP10*, *DAPK3*, *GNA11*, *CTNNAL 1*, *OPTN*, *CYR61*, *EGFR*, *BCAM*, *APLP2*, and *CAV1*), among which some may participate in tumor growth and directly or indirectly contribute to cancer occurrence.

MM is a plasma cell proliferative disorder inducing 1% of all cancers and 10% of hematological malignancies [5,6]. SAF exhibited indispensable cytotoxicity against different cell lines with special interest in NCI-H929 cells from MM cells. SAF inhibited microtubule formation and induced S and G2/M arrest and apoptosis in those cells. SAF may be a convincing candidate in MM therapy, but needs further investigation to determine its therapeutic index.

## 4. Materials and Methods

### 4.1. Cell Lines

Leukemia cells including CCRF-CEM, HL-60, MOLT-4, and NB-4 as well as MM cells comprising KMS-12BM, KMS-11, MOLP-8, NCI-H929, L-363, RPMI-8226, AMO-I, JJN-3, and OPM-2 were cultured in Roswell Park Memorial Institute (RPMI) 1640 medium, supplemented with 10% fetal bovine serum (FBS) (Invitrogen, Darmstadt, Germany) and 1% penicillin (100 U/mL)-streptomycin (100 µG/mL) (PIS) antibiotic (Invitrogen) and incubated in humidified 5% CO_2_ atmosphere at 37 °C. Cells were passaged twice weekly. All experiments were conducted with the cells in their logarithmic growth phase.

U2OS human osteosarcoma cancer cells, stably transfected with an α-tubulin-GFP construct, were cultured in Dulbecco’s Modified Eagle Medium (DMEM) medium with 10% FBS and 1% penicillin (100 U/mL)-streptomycin (100 µG/mL) (PIS) antibiotic (Invitrogen) and continuously treated with 250 µg/mL geneticin at 37 °C and 5% CO_2_ to maintain α-tubulin expression.

A panel of 59 human tumor cell lines of the Developmental Therapeutics Program of the NCI (Bethesda, MD, USA) contains carcinoma, prostate carcinoma, leukemia, melanoma, breast, colon, renal, ovarian, and non-small cell lung cancers as well as tumor cells of the central nervous system [54]. Cells treated with secalonic acid and cytotoxicity was assessed by the sulforhodamine B assay [55].

### 4.2. Cytotoxicity Assay

SAF was obtained from MicroCombiChem (Wiesbaden, Germany). The compound had a purity of ≥95% (HPLC). The cytotoxicity of SAF was studied by the use of the resazurin reduction assay [56,57]. The assay is based on the reduction of resazurin to resorufin by viable cells. Non-viable cells do not exhibit blue staining due to losing their metabolic capacity. Briefly, 1 × 10^4^ cells in a total volume of 100 μL were seeded in a 96-well cell culture plate. The cells were incubated with various concentrations of SAF to obtain a total volume of 200 μL/well for 72 h. Then, 0.01% of resazurin (Sigma-Aldrich, Schnelldorf, Germany) diluted in double-distilled water (ddH2O) was added (20 μL/well) and incubated for another 4 h. Infinite M2000 Pro^TM^ plate reader (Tecan, Crailsheim, Germany) was used to measure the fluorescence using an excitation wavelength of 544 nm and an emission wavelength of 590 nm. Each assay was independently performed at least three times, with six parallel replicates each. Dose response curves of each cell were formed using GraphPad Prism^®^ v6.0 software (GraphPad Software Inc., San Diego, CA, USA). The concentrations required for 50% inhibition (IC_50_) were calculated by nonlinear regression using Microsoft Excel.

### 4.3. Toxicity of Secalonic Acid F in Healthy Cells

Fresh peripheral blood samples were obtained from healthy donors and transferred into plastic Monovette EDTA tubes. The human peripheral mononuclear cells (PMNC) were isolated by Histopaque^®^ (Sigma-Aldrich, St. Louis, MO, USA), according to the previously described method [58]. Briefly, 3 mL blood was added carefully over 3 mL of Histopaque^®^ and centrifuged at 400× *g* for 30 min at 4 °C. Subsequently, the layer containing lymphocytes and PMNC was transmitted into a new tube and washed several times with PBS. Isolated cells were then maintained in a Panserin 413 medium (PAN-Biotech, Aidenbach, Germany) supplemented with 2% phytohemagglutinin M (PHA-M, Life Technologies, Darmstadt, Germany). Finally, the resazurin assay was performed to assess cell viability as described above.

### 4.4. Analysis of Cell Cycle Distribution by Flow Cytometry

The NCI-H929 cells (1 × 10^6^ cells/well) were seeded into 6-well plates and treated with serial dilutions of secalonic acid including 0.5 × IC_50_, IC_50_, 2 × IC_50_, and 4 × IC_50_ for 24 and 48 h. The cells were collected, washed with PBS, and fixed with 96% ice-cold ethanol. After fixation, the cells were washed with PBS again, dissolved in PBS, and stained with propidium iodide (PI, Sigma-Aldrich) at a final concentration of 50 μg/mL for 15 min at room temperature in the dark. Cell cycle analyses were performed using a BD Accuri™ C6 Flow cytometer (Becton-Dickinson, Heidelberg, Germany) at 488 nm excitation wavelength, and emission was measured by a 610/20 nm bandpass filter. A total number of 1 × 10^4^ cells were counted for each experiment. All experiments were performed at least in triplicate.

### 4.5. Detection of Apoptosis/Necrosis by Annexin V/PI Staining

The NCI-H929 cells (1 × 10^6^ cells/well) were treated with secalonic acid for 24 h and 48 h (in humidified 5% CO_2_ atmosphere at 37 °C), and apoptosis/necrosis was assessed by the flouresceinisothiocynate (FITC)-conjugated annexin V/PI assay kit (eBioscience™Annexin V; Invitrogen, Germany) by flow cytometry. Briefly, treated cells were centrifuged at 1200 rpm for 5 min and washed twice with ice-cold PBS (phosphate-buffered saline) and 1 × binding buffer, respectively. After the cells were re-suspended in binding buffer at a total number of 4 × 10^6^ cells/mL, 5 µL of FITC-conjugated annexin V was added to 100 µL of the cell suspension and incubated for 15 min at room temperature. Then, the cells were washed with 1 × binding buffer and resuspended in 200 µL of 1 × binding buffer. Propidium iodide staining solution (5 µL) was added and analyzed after 15 min incubation at room temperature in the dark using BD Accury C6 Flow Cytometer (BD Biosciences). Apoptosis and necrosis were evaluated on fluorescence 2 (FL3 for propidium iodide) versus fluorescence 1 (FL1 for annexin) plots. The fluorescent cells % in each quadrant pointed out different cell populations corresponding to viable and non-apoptotic (annexin V−PI−), early apoptotic (annexin V+PI−), late apoptotic as well as early necrotic (annexin V+PI+) and late necrotic (annexin V−PI+) cells.

### 4.6. COMPARE and Hierarchical Cluster Analyses of Microarray Data

Messenger RNA expression profiles of 59 human cancer cell lines were deposited at the database of the DTP of the NCI (http://dtp.nci.nih.gov). The COMPARE analysis, a web-based algorithm, associates transcriptome-wide mRNA expressions to drug response of the NCI cell line panel (https://dtp.cancer.gov) based on Pearson’s rank correlation coefficient [59]. We executed the COMPARE analyses between the IC_50_ values for secalonic acid and the microarray-based transcriptome-wide mRNA expression levels in the NCI cell lines to detect the genes, which were related to their sensitivity or resistance to secalonic acid, respectively.

Subsequently, we performed a hierarchical cluster analysis (WARD method) to cluster the mRNA expressions of genes determined via COMPARE analysis by the CIMMINER program (https://discover.nci.nih.gov/cimminer/) and the heatmap was formed accordingly.

Pearson’s correlation test calculated the significance values (*p*-values) and ranked correlation coefficients (*R*-values). The median log_10_IC_50_ value was considered as a cut-off threshold deciding whether cell lines were sensitive or resistant to secalonic acid. The chi -square (χ^2^) test was performed to verify bivariate frequency distributions for pairs of nominal scaled variables for dependencies gained from cluster analysis/heat mapping.

### 4.7. Imaging of Structure and Dynamics of the Microtubule Cytoskeleton by Fluorescence Microscopy

U2OS-GFP -α-tubulin cells (5 × 10^5^/well) were seeded into 6-well plates, each including a sterile ibi Treat μ-slide (ibidi, Germany). The cells were allowed to attach overnight, treated with 40 µM of SAF or DMSO (solvent control) and incubated at 37 °C for 24 h. Then, the cells were rinsed with PBS and fixed by 4% *p*-formaldehyde at room temperature for 30 min. Subsequent to the washing with PBS and staining for 5 min with 1 µM of 4′,6-diamidino-2-phenylindole (DAPI) (Life Technologies, Darmstadt, Germany), the cells were washed with PBS again and mounted. Fluorescence imaging was performed by using 470 nm excitation and 525 nm emission for GFP and 447 nm emission for DAPI with an EVOS digital inverted microscope (Life Technologies). Each experiment was done at least in triplicate and representative images were selected.

## 5. Conclusions

In conclusion, in the present study SAF was tested for the first time against several multiple myeloma cells with a focus on determining its mechanism of action. We integrated various computational approaches into the in vitro experimental tests, which enabled validation of in silico analyses by experimental data. Our findings demonstrated that SAF remarkably inhibited the growth of NCI-H929 cells. These effects were related to the cell cycle arrest of the G2/M phase, apoptosis, necrosis, and the suppression of microtubule formation. Furthermore, it did not reveal considerable toxicity towards normal cells. Computational analyses further point out that possible targets associated with cell growth, migration, and invasion may be involved in SAF’s cytotoxicity. This study not only renders a better understanding of how SAF acts as an antitumor agent on MM cells, but also provides a possible role of this drug in the treatment of MM.

## Figures and Tables

**Table 1:** COMPARE analysis of genes, whose microarray-based mRNA expression correlated with log_10_IC_50_ values for secalonic acid in a panel of 59 cell lines.

R Value	Gene Symbol	Genbank Accession Number	Pattern ID	Gene Name	Gene Function
**Standard COMPARE (Resistance Genes)**					
0.503	*PIKFYVE*	AB023198	GC35064	Phosphoinositide kinase, FYVE finger containing	Required for endocytic-vacuolar pathway and nuclear migration
0.502	*NDUFS1*	X61100	GC28806	NADH dehydrogenase (ubiquinone) Fe-S protein (NADH-coenzyme Q reductase)	Core subunit of the mitochondrial membrane respiratory chain
0.499	*PIGF*	D13435	GC32669	Phosphatidylinositol glycan anchor biosynthesis, class F	Involved in GPI-anchor biosynthesis
0.499	*NUP50*	N42007	GC30995	Nucleoporin	Component of the nuclear pore complex that has a direct role in nuclear protein import
0.481	*HADHB*	D16481	GC30165	Hydroxyacyl-CoA dehydrogenase/3-ketoacyl-CoA thiolase/enoyl-CoA hydratase (trifunctional protein), b	Encodes a protein, which catalyzes the last three steps of mitochondrial beta-oxidation of long chain fatty acids
0.474	*RQCD1*	AA160724	GC36841	RCD1 required for cell differentiation1 homolog (*S. pombe*)	Involved in various cellular processes including bulk mRNA degradation, miRNA-mediated repression, translational repression and general transcription regulation
0.474	*PLCL1*	D42108	GC33166	Phospholipase C-like 1	Involved in an inositol phospholipid-based intracellular signaling cascade. Regulates the turnover of receptors and contributes to the maintenance of GABA-mediated synaptic inhibition
0.468	*GK*	L13943	GC28538	Glycerol kinase	Key enzyme in the regulation of glycerol uptake and metabolism
0.454	*AP3M2*	D38293	GC27638	Adaptor-related protein complex 3, µ2 subunit	Involved in the budding of vesicles from the Golgi membrane and trafficking to lysosomes
0.453	*IRF2*	X15949	GC33202	Interferon regulatory factor 2	Involved in cell cycle regulation and antagonizes IRF1 transcriptional activation
0.45	*CTRL*	X71877	GC30632	Chymotrypsin-like	Encodes a serine-type endopeptidase with chymotrypsin- and elastase-2-like activities. The gene encoding this zymogen is expressed specifically in the pancreas and likely functions as a digestive enzyme
0.449	*ATP5G3*	U09813	GC37818	ATP synthase, H^+^ transporting, mitochondrial Fo complex, subunit C3 (subunit 9)	Produces ATP from ADP in the presence of a proton gradient across the membrane
0.448	*SF3B1*	AF054248	GC29865	Splicing factor 3b, subunit 1	Required for A complex assembly and involved in the assembly of the E complex
0.446	*TNRC6B*	AB029016	GC27890	Trinucleotide repeat containing 6B	Plays a role in RNA-mediated gene silencing and is required for miRNA-dependent translational repression and siRNA-dependent endonucleolytic cleavage of complementary mRNAs by argonaute family proteins
0.445	*HOMER1*	Y17829	GC31446	Homer homolog 1 (*Drosophila*)	Involved in the regulation the trafficking and surface expression of GRM5 and the structural changes that occur at synapses during long-lasting neuronal plasticity and development
0.445	*USP34*	AB018272	GC31656	Ubiquitin specific peptidase 34	Regulates Wnt signaling pathway and involved in the processing of poly-ubiquitin precursors as well as that of ubiquitinated proteins
0.444	*BLM*	U39817	GC33552	Bloom syndrome, RecQ helicase-like	Participates in DNA replication and repair
0.443	*IDH3A*	U07681	GC39215	Isocitrate dehydrogenase 3 (NAD^+^) α	Catalyze the oxidative decarboxylation of isocitrate to 2-oxoglutarate
0.441	*ZKSCAN1*	U09848	GC32155	Zinc finger with KRAB and SCAN domains 1	Regulates the expression of GABA type-A receptors in the brain
0.436	*NDUFA6*	AI223047	GC29399	NADH dehydrogenase (ubiquinone) 1 α subcomplex 6	Accessory subunit of the mitochondrial membrane respiratory chain NADH dehydrogenase (Complex I)
**Reverse COMPARE (Sensitive Genes)**					
−0.567	*APBB2*	AL080130	GC30961	Amyloid beta (A4) precursor protein-binding, family B, member 2	Interacting with the cytoplasmic domains of amyloid beta (A4) precursor protein and amyloid beta (A4) precursor-like protein 2 involved in signal transduction
−0.548	*ITGA3*	M59911	GC32775	Integrin, α3 (antigen CD49C, α3 subunit of VLA-3 receptor)	Participates in the adhesion, formation of invadopodia and matrix degradation processes, promoting cell invasion
−0.532	*TRIP10*	AJ000414	GC39136	Thyroid hormone receptor interactor 10	Required for translocation of GLUT4 to the plasma membrane and coordinating membrane tubulation with reorganization of the actin cytoskeleton
−0.514	*DAPK3*	AB007144	GC36901	Death-associated protein kinase 3	Regulates apoptosis, autophagy, transcription, translation and actin cytoskeleton reorganization
−0.506	*GNA11*	N36926	GC31915	Guanine nucleotide binding protein (G protein), α11 (Gq class) RNA	Functioning as modulators or transducers in various transmembrane signaling systems
−0.505	*NR2F2*	M64497	GC29817	Nuclear receptor subfamily 2, group F, member 2	Regulates the apolipoprotein A-I gene transcription
−0.501	*PGRMC2*	AJ002030	GC29236	Progesterone receptor membrane component 2	Receptor for steroids. Up-regulated during the secretory phase of the menstrual cycle.
−0.489	*CTNNAL1*	U97067	GC38343	Catenin (cadherin-associated protein), α-like 1	Modulates the Rho pathway signaling by providing a scaffold for the Lbc Rho guanine nucleotide exchange factor (ARHGEF1)
−0.488	*OPTN*	AF070533	GC32186	Optineurin	Plays an important role in the maintenance of the Golgi complex, in membrane trafficking, in exocytosis, through its interaction with myosin VI and Rab8.
−0.488	*CNN3*	S80562	GC31388	Calponin 3, acidic	Regulates and modulates smooth muscle contraction
−0.486	*PEA15*	X86809	GC35241	Phosphoprotein enriched in astrocytes 15	Blocks Ras-mediated inhibition of integrin activation and modulates the ERK MAP kinase cascade. Inhibits RPS6KA3 (Ribosomal Protein S6 Kinase A3), CASP8 activities and apoptosis and regulates glucose transport
−0.486	*CYR61*	Y11307	GC29186	Cysteine-rich, angiogenic inducer, 61	Promotes cell proliferation, chemotaxis, angiogenesis and cell adhesion
−0.484	*EGFR*	X00588	GC33544	Epidermal growth factor receptor	Activates several signaling cascades such as RAS-RAF-MEK-ERK, PI3 kinase-AKT to convert extracellular cues into appropriate cellular responses
−0.484	*MXRA7*	AL046940	GC31711	Matrix-remodeling associated 7	Acts on tissue remodeling and may be associated with diseases like endometriosis of ovary
−0.479	*PLIN3*	AF057140	GC30596	Perilipin 3	Required for the transport of mannose 6-phosphate receptors
−0.479	*MYOF*	AL096713	GC37683	Myoferlin	Plays a role in the plasmalemma repair mechanism of endothelial cells Involved in endocytic recycling and VEGF signal transduction
−0.476	*CAPN2*	M23254	GC27400	Calpain 2, (m/II) large subunit	Involved in cytoskeletal remodeling and signal transduction
−0.472	*BCAM*	X83425	GC30519	Basal cell adhesion molecule (Lutheran blood group)	Mediate intracellular signaling and play a role in epithelial cell cancer and in vaso-occlusion of red blood cells in sickle cell disease
−0.471	*APLP2*	S60099	GC36942	Amyloid β (A4) precursor-like protein 2	Play a role in the regulation of hemostasis
−0.467	*CAV1*	AF070648	GC39139	Caveolin 1, caveolae protein	Regulates G-protein activity

**Table 2:** Separation of clusters of NCI cell lines obtained by hierarchical cluster analysis for secalonic acid.

Secalonic Acid	Sensitive	Resistant
Partition (log_10_IC_50_)	<−7.03 M	≥−7.03 M
Cluster 1	8	1
Cluster 2	10	3
Cluster 3	3	15
Cluster 4	7	11
χ^2^ test	*p* = 4.29 × 10^−4^	

The median log_10_IC_50_ value (M) for secalonic acid was used as a cut-off separate cancer cell lines as being “sensitive” or “resistant”.

## References

[1] World Health Organization. https://www.who.int/news-room/fact-sheets/detail/cancer.

[2] Abe J.-I., Martin J.F., Yeh E.T.H. (2016). The future of onco-cardiology: We are not just “side effect hunters”. Circ. Res..

[3] Tewari D., Rawat P., Singh P.K. (2019). Adverse drug reactions of anticancer drugs derived from natural sources. Food Chem. Toxicol..

[4] Henning R.J., Harbison R.D. (2017). Cardio-oncology: Cardiovascular complications of cancer therapy. Future Cardiol..

[5] Rajkumar S.V., Dimopoulos M.A., Palumbo A., Blade J., Merlini G., Mateos M.V., Kumar S., Hillengass J., Kastritis E., Richardson P. (2014). International myeloma working group updated criteria for the diagnosis of multiple myeloma. Lancet Oncol..

[6] Vincent R.S. (2016). Multiple myeloma: 2016 update on diagnosis, risk-stratification, and management. Am. J. Hematol..

[7] Palumbo A., Anderson K. (2011). Multiple myeloma. N. Engl. J. Med..

[8] Brenner H., Gondos A., Pulte D. (2008). Recent major improvement in long-term survival of younger patients with multiple myeloma. Blood.

[9] Lida S. (2016). Mechanisms of action and resistance for multiple myeloma novel drug treatments. Int. J. Hematol..

[10] Nass J., Efferth T. (2018). Drug targets and resistance mechanisms in multiple myeloma. Cancer Drug Resist..

[11] Kumar S.K., Dispenzieri A., Lacy M.Q., Gertz M.A., Buadi F.K., Pandey S., Kapoor P., Dingli D., Hayman S.R., Leung N. (2014). Continued improvement in survival in multiple myeloma: Changes in early mortality and outcomes in older patients. Leukemia.

[12] Juliusson G., Hough R. (2016). Leukemia. Prog. Tumor. Res..

[13] National Cancer Institute. https://www.cancer.gov/types/leukemia.

[14] Terwilliger T., Abdul-Hay M. (2017). Acute lymphoblastic leukemia: A comprehensive review and 2017 update. Blood Cancer J..

[15] Jabbour E., O’Brien S., Konopleva M., Kantarjian H. (2015). New insights into the pathophysiology and therapy of adult acute lymphoblastic leukemia. Cancer.

[16] Döhner H., Estey E.H., Amadori S., Appelbaum F.R., Büchner T., Burnett A.K., Dombret H., Fenaux P., Grimwade D., Larson R.A. (2010). Diagnosis and management of acute myeloid leukemia in adults: Recommendations from an international expert panel, on behalf of the European LeukemiaNet. Blood.

[17] Newman D.J., Cragg G.M. (2016). Natural products as sources of new drugs from 1981 to 2014. J. Nat. Prod..

[18] Yamazaki M., Maebayashi Y., Miyaki K. (1971). The isolation of secalonic acid A from *Aspergillus ochraceus* cultured on rice. Chem. Pharm. Bull..

[19] Andersen R., Buechi G., Kobbe B., Demain A.L. (1977). Secalonic acids D and F are toxic metabolites of Aspergillus aculeatus. J. Org. Chem..

[20] Lazarovits G., Steele R.W., Higgins V.J., Stoessl A. (1989). Tricyclazole as an inhibitor of polyketide metabolism in the onion pink root rot pathogen, Pyrenochaeta terrestris. Pestic. Biochem. Phys..

[21] Luisa B.G. (2012). Handbook of Toxic Fungal Metabolites.

[22] Steyn P.S. (1970). The isolation, structure and absolute configuration of secalonic acid D, the toxic metabolite of Penicillium oxalicum. Tetrahedron.

[23] Betina V. (1984). Mycotoxins: Production, Isolation, Separation, and Purification.

[24] Zhang W., Krohn K., Ullah Z., Flörke U., Pescitelli G., Di Bari L., Antus S., Kurtán T., Rheinheimer J., Draeger S. (2008). New mono- and dimeric members of the secalonic acid family: Blennolides A–G isolated from the fungus *Blennoria* sp.. Chemistry.

[25] Guo Z., She Z., Shao C., Wen L., Liu F., Zheng Z., Lin Y. (2007). 1H and 13C NMR signal assignments of paecilin A and B, two new chromone derivatives from mangrove endophytic fungus Paecilomyces sp. (tree 1–7). Magn. Reson. Chem..

[26] Guru S.K., Pathania A.S., Kumar S., Ramesh D., Kumar M., Rana S., Kumar A., Malik F., Sharma P.R., Chandan B.K. (2015). Secalonic acid-d represses hif1α/vegf-mediated angiogenesis by regulating the Akt/mTOR/p70S6K signaling cascade. Cancer Res..

[27] Harada M., Yano S., Watanabe H., Yamazaki M., Miyaki K. (1974). Phlogistic activity of secalonic acid A. Chem. Pharm. Bull.

[28] Iwaguchi T., Kitagawa H., Hirose K., Ishida T., Yamamoto T. (1980). 5-di-(2′-tetrahydropyranyl)secalonic acid D as a new antibiotic derivative with anticancer activity. GANN.

[29] Kurobane I., Iwahashi S., Fukuda A. (1987). Cytostatic activity of naturally isolated isomers of secalonic acids and their chemically rearranged dimers. Drugs Exp. Clin. Res..

[30] Shimizu M., Nakamura M., Kataoka T., Iwaguchi T. (1983). Mechanism of the antitumor activity of 5,5′-bis(2′-tetrahydropyranyl) secalonic acid D against Meth-A. Cancer Chemother. Pharmacol..

[31] Cai X.L., Gao J.P., Li Q., Wen L., She Z.G., Lin Y.C. (2008). Cytotoxicity of the secondary metabolites of marine mangrove fungus Paecilomyces sp. tree 1-7 on human hepatoma cell line HepG2. Zhong Yao Cai.

[32] Hong R. (2011). Secalonic acid D as a novel DNA topoisomerase I inhibitor from marine lichen-derived fungus Gliocladium sp. T31. Pharm. Biol..

[33] Ren H., Tian L., Gu Q., Zhu W. (2006). Secalonic acid D.; A cytotoxic constituent from marine lichen-derived fungus Gliocladium sp. T31. Arch. Pharm. Res..

[34] Tang R., Kimishima A., Setiawan A., Arai M. (2020). Secalonic acid D as a selective cytotoxic substance on the cancer cells adapted to nutrient starvation. J. Nat. Med..

[35] Hu Y.P., Tao L.Y., Wang F., Zhang J.Y., Liang Y.J., Fu L.W. (2013). Secalonic acid D reduced the percentage of side populations by down-regulating the expression of ABCG2. Biochem. Pharmacol..

[36] Liao G., Zhou J., Wang H., Mao Z., Xiao W., Wang H., She Z., Zhu Y. (2010). The cell toxicity effect of secalonic acid D on GH3 cells and the related mechanisms. Oncol. Rep..

[37] Zhang J.Y., Tao L.Y., Liang Y.J., Yan Y.Y., Dai C.L., Xia X.K., She Z.G., Lin Y.C., Fu L.W. (2009). Secalonic acid D induced leukemia cell apoptosis and cell cycle arrest of G(1) with involvement of GSK-3beta/beta-catenin/c-Myc pathway. Cell Cycle.

[38] Zhang H., Huang L., Tao L., Zhang J., Wang F., Zhang X., Fu L. (2019). Secalonic acid D induces cell apoptosis in both sensitive and ABCG2-overexpressing multidrug resistant cancer cells through upregulating c-Jun expression. Acta Pharm. Sin. B..

[39] Cherigo L., Lopez D., Martinez-Luis S. (2015). Marine natural products as breast cancer resistance protein inhibitors. Mar. Drugs.

[40] Aberhart D., Chen Y., De Mayo P., Stothers J. (1965). Mould metabolites—IV: The isolation and constitution of some ergot pigments. Tetrahedron.

[41] Zeng R., Luo S., Shi M., Shi Y., Zeng Q., Tan H. (2001). Allelopathy of Aspergillus japonicus on crops. Agron. J..

[42] Zeng R.S., Luo S.M., Shi Y.H., Shi M.B., Tu C.Y. (2001). Physiological and biochemical mechanism of allelopathy of secalonic acid F on higher plants. Agron. J..

[43] Gao X., Sun H.L., Liu D.S., Zhang J.R., Zhang J., Yan M.M., Pan X.H. (2017). Secalonic acid- F inhibited cell growth more effectively than 5-fluorouracil on hepatocellular carcinoma in vitro and in vivo. Neoplasma.

[44] Xie L., Li M., Liu D., Wang X., Wang P., Dai H., Yang W., Liu W., Hu X., Zhao M. (2019). Secalonic acid-F, a novel mycotoxin, represses the progression of hepatocellular carcinoma via MARCH1 regulation of the PI3K/AKT/β-catenin signaling pathway. Molecules.

[45] Li N., Yi Z., Wang Y., Zhang Q., Zhong T., Qiu Y., Wu Z., Tang X. (2012). Differential proteomic analysis of HL60 cells treated with secalonic acid F reveals caspase 3-induced cleavage of Rho GDP dissociation inhibitor 2. Oncol. Rep..

[46] Reddy C.S., Hayes A.W., Williams W.L., Ciegler A. (1979). Toxicity of secalonic acid D. J. Toxicol. Environ. Health.

[47] Ross D.T., Scherf U., Eisen M.B., Perou C.M., Rees C., Spellman P., Iyer V., Jeffrey S.S., Van de Rijn M., Waltham M. (2000). Systematic variation in gene expression patterns in human cancer cell lines. Nat. Genet..

[48] Scherf U., Ross D.T., Waltham M., Smith L.H., Lee J.K., Tanabe L., Kohn K.W., Reinhold W.C., Myers T.G., Andrews D.T. (2000). A gene expression database for the molecular pharmacology of cancer. Nat. Genet..

[49] Weinstein J.N. (1998). Fishing expeditions. Science.

[50] Dumontet C., Jordan M.A. (2010). Microtubule-binding agents: A dynamic field of cancer therapeutics. Nat. Rev. Drug Discov..

[51] Jordan M.A., Wilson L. (2004). Microtubules as a target for anticancer drugs. Nat. Rev. Cancer.

[52] Oshiro C., Marsh S., McLeod H., Carrillo M.W., Klein T., Altman R. (2009). Taxane pathway. Pharmacogenet. Genom..

[53] Kaverina I., Straube A. (2011). Regulation of cell migration by dynamic microtubules. Semin. Cell Dev. Biol..

[54] Alley M.C., Scudiero D.A., Monks A., Hursey M.L., Czerwinski M.J., Fine D.L., Abbott B.J., Mayo J.G., Shoemaker R.H., Boyd M.R. (1988). Feasibility of drug screening with panels of human tumor cell lines using a microculture tetrazolium assay. Cancer Res..

[55] Rubinstein L.V., Shoemaker R.H., Paull K.D., Simon R.M., Tosini S., Skehan P., Scudiero D.A., Monks A., Boyd M.R. (1990). Comparison of in vitro anticancer-drug-screening data generated with a tetrazolium assay versus a protein assay against a diverse panel of human tumor cell lines. J. Natl. Cancer Inst..

[56] Kuete V., Mbaveng A.T., Nono E.C., Simo C.C., Zeino M., Nkengfack A.E., Efferth T. (2016). Cytotoxicity of seven naturally occurring phenolic compounds towards multi-factorial drug-resistant cancer cells. Phytomedicine.

[57] O’Brien J., Wilson I., Orton T., Pognan F. (2000). Investigation of the Alamar Blue (resazurin) fluorescent dye for the assessment of mammalian cell cytotoxicity. Eur. J. Biochem..

[58] Saeed M.E.M., Mahmoud N., Sugimoto Y., Efferth T., Abdel-Aziz H. (2018). Molecular determinants of sensitivity or resistance of cancer cells toward sanguinarine. Front. Pharmacol..

[59] Paull K.D., Shoemaker R.H., Hodes L., Monks A., Scudiero D.A., Rubinstein L., Plowman J., Boyd M.R. (1989). Display and analysis of patterns of differential activity of drugs against human tumor cell lines: Development of mean graph and COMPARE algorithm. J. Natl. Cancer Inst..

